# AEF: Adaptive En-Route Filtering to Extend Network Lifetime in Wireless Sensor Networks

**DOI:** 10.3390/s19184036

**Published:** 2019-09-19

**Authors:** Muhammad K. Shahzad, S. M. Riazul Islam, Kyung-Sup Kwak, Lewis Nkenyereye

**Affiliations:** 1 Department of Computing, SEECS, National University of Sciences and Technology, Islamabad 44000, Pakistan; mkhuram.shahzad@seecs.edu.pk; 2 Department of Computer Science and Engineering, Sejong University, Seoul 05006, Korea; riaz@sejong.ac.kr; 3 Department of Information and Communication Engineering, Inha University, Incheon 22212, Korea; 4 Department of Computer and Information Security, Sejong University, Seoul 05006, Korea

**Keywords:** filtering capacity, adaptive filtering, energy-aware routing, network lifetime, energy efficiency, wireless sensor networks

## Abstract

Static sink-based wireless sensor networks (WSNs) suffer from an energy-hole problem. This incurs as the rate of energy consumption on sensor nodes around sinks and on critical paths is considerably faster. State-of-the-art en-routing filtering schemes save energy by countering false report injection attacks. In addition to their unique limitations, these schemes generally do not examine energy awareness in underlying routing. Mostly, these security methods are based on a fixed filtering capacity, unable to respond to changes in attack intensity. Therefore, these limitations cause network partition(s), exhibiting adverse effects on network lifetime. Extending network lifetime while preserving energy and security thus becomes an interesting challenge. In this article, we address the aforesaid shortcomings with the proposed adaptive en-route filtering (AEF) scheme. In energy-aware routing, the fitness function, which is used to select forwarding nodes, considers residual energy and other factors as opposed to distance only. In pre-deterministic key distribution, keys are distributed based on the consideration of having paths with a different number of verification nodes. This, consequently, permits us to have multiple paths with different security levels that can be exploited to counter different attack intensities. Taken together, the integration of the special fitness function with the new key distribution approach enables the AEF to adapt the underlying dynamic network conditions. The simulation experiments under different settings show significant improvements in network lifetime.

## 1. Introduction

Wireless sensor networks (WSNs) have witnessed mushroom growth due to the recent advancement in microelectromechanical systems (MEMS) [1,2]. WSNs are being adapted in numerous applications due to their low cost, small size, tether-lessness, and high density characteristics. Their widespread deployment requires countermeasures against security threats. One such threat is false report injection attacks, hereinafter referred to as attacks, resulting in draining energy of nodes on the path.

**Figure 1:**
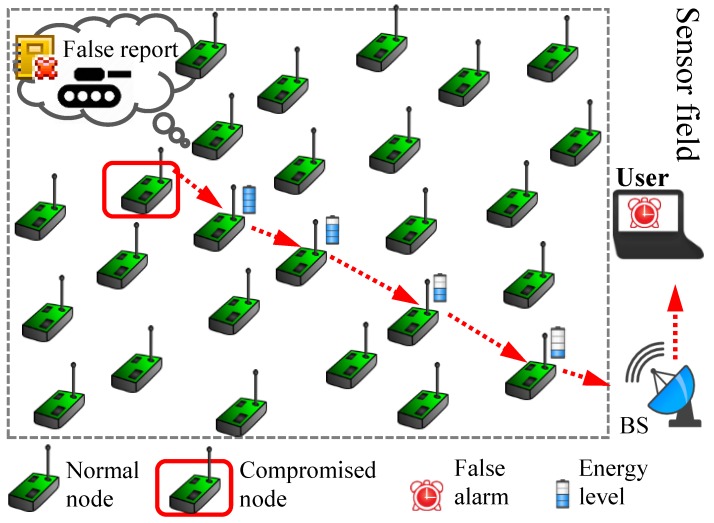
False report injection attack in wireless sensor networks.

An example of such an attack is shown in Figure 1. It is worth mentioning that, although our proposed scheme can also be effective against eavesdropping or modification attacks, this is out of the scope of our paper. A compromised node generates a false report about a nonexistent event and transmits it to the base station (BS), draining energy of the nodes on the path. It could also trigger false alarms about nonexistent events to the end user. En-route filtering schemes in general and dynamic en-route filtering (DEF) [3] and commutative cipher-based en-route filtering (CCEF) [4] in particular offer better security at the expense of network lifetimes. DEF uses the hill-climbing approach for key dissemination for early detection of false report attacks. A legitimate report is approved by several nodes using their keys and secret key from a global key poll. Verification nodes on path can drop reports which are not validated. Since, DEF uses many keys in a key chain method, it becomes complicated for large-size WSNs. On the other hand, CCEF is based on expansive public key infrastructure.

In most en-route filtering schemes [3,4,5,6,7,8], similar limitations are observed: (1) an underlying fixed path routing that does not consider network lifetime in the design and (2) fixed responses (i.e., detection capacity) to different number of attacks, among other limitations. An example of shortest path or greedy routing, which results in fixed path routing, is greedy perimeter-based stateless routing (GPSR) [9]. In this paper, the first limitation is countered by the proposed energy-aware dynamic routing and the other is catered by pre-deterministic key distribution. These methods equip our proposed scheme to dynamically respond to network conditions and attack intensity.

Dynamic path routing not only serves our purpose of avoiding the energy-hole problem but also can grant multiple paths and load balancing. Sensor networks suffers from the energy-hole problem as the rate of energy consumption on critical paths and around the base station is significantly higher compared to the distant nodes. It results in network partition—communication between different parts of the network is broken. This is such a state at which disconnected parts of network cannot be reached. Therefore, the energy-hole problem resulting in network partitioning can be avoided using dynamic path routing.

In this paper, we prefer dynamic path routing over other strategies which may alleviate energy-hole problems such as dynamic clustering and sink mobility. Compared to the aforementioned techniques, the dynamic routing strategy grants additional multiple paths with different number of keys in each path along with a distinct level of security. As a result, our scheme can respond dynamically with changes in attack intensity and load balancing while avoiding the energy-hole problem.

In addition to countering attacks, the proposed scheme can also be effective against eavesdropping or modification attacks [10]. Since, event reports are not transmitted through a single path, an adversary cannot interpret the entire message. As events report are routed through multiple paths, AEF can be more robust and reliable against these attacks.

Various assumptions about radio characteristics might be beneficial to different protocols such as energy dissipated in transmitters and receivers. Here, we assume a simple and most commonly used radio model [11] to achieve an acceptable $E_{b}/N_{0}$ [12]. En-route filtering schemes, notably, DEF and CCEF, result in a better security at the expense of network lifetime. Reference [13] has proposed to increase network lifetime by improving routing. There is a need to extend network lifetime in filtering schemes which often ignores underlying routing. Additionally, we proposed pre-deterministic key distribution, dynamic path routing, and dynamic response to attack frequency changes instead of a fixed probabilistic approach.

A number of research works [14,15,16,17,18,19,20] emphasize attacks and countermeasures. In the past, energy-hole problem mitigation was discussed in Reference [21]. MICA2 [22] sensor motes sensing platform was used in the experiments. Sensor clocks are synchronised using the energy-efficient time synchronization protocol (ETSP) [23]. Recent work [20,24,25,26,27] also highlighted countermeasures against attacks in en-route filtering with an emphasis on network lifetime. Several work focused on network lifetime extension [10,28,29,30,31], energy efficiency and network lifetime [32,33,34], and energy efficiency [35,36,37,38,39] and privacy [40,41,42] using various techniques.

In this paper, we aim to improve network lifetime without compromising detection capacity and energy efficiency in en-route filtering schemes. The proposed scheme is adaptive since it is based on energy-aware dynamic path routing and has the ability to counter variation in attacks on WSNs.

Simulation experiments validate the efficacy of our scheme. AEF shows an average of 2.64 times (50% false traffic ratio (FTR)) and 2.63 folds (70% FTR and 2.61 (90%). In comparison with CCEF, we observe averages of 1.14 (50%), 1.10 (70%), and 1.05 (90%) times gain. The detection capacity and energy-efficiency performance are similar to the existing schemes. Our main contribution in this work are as follows:

- Introducing dynamic security response to counter attack-intensity variations, i.e., higher security for larger attacks and vice versa. This is achieved by pre-deterministic key-distribution which allows different paths with a variable number of verification nodes and hence security.
- Proposing energy-aware dynamic routing that helps load balancing over a larger group of participating nodes.
- Mitigating the energy hole and significantly extending network lifetime by using the aforementioned methods.
- In summary, despite a reasonable volume of related research to improve network lifetime in the routing layer, this work focuses on prolonging network lifetime in security schemes.

## 2. Related Work

Dynamic en-route filtering (DEF) [3], uses the hill-climbing approach to distribute keys and effectively counters attacks to save scarce energy. Each node transmits its key to the forwarding node. Sending nodes reveal their keys after sending reports, enabling the forwarding node (s) to validate their reports. It utilizes the broadcasting nature of wireless networks to defend against denial of service (DoS) attacks and multipath routing to counter topology changes. DEF can counter attacks requiring lower memory as compared to other schemes.

Statistical en-route filtering (SEF) [5] was the first work to detect fabricated reports. A global key pool is split into nonoverlapping segments. A small number of keys is preloaded on each node before deployment. Each event detecting node generates a message authentication code (MAC) using one of its stored keys. Event detection nodes elect a center of stimulus (CoS) node to collect and summarize all the received detection results to produce a report including the respective MACs. A report with an inadequate number of MACs will not be forwarded. False reports reached at the base station (BS) will be finally detected since BS has all the keys. SEF has only a limited filtering capacity and fails to counter impersonating attacks.

Commutative Cipher-based En-route Filtering (CCEF) [4] saves energy by early detection. It has the following limitations: fix path routing, not considering residual energy, and fix detection capacity. It does not respond according to the change of attack frequency. Underlying fixed path routing is not energy aware so it exhibits adverse effects on network lifetime. Secure ticket-based en-route filtering (STEF) [6] uses tickets issued by a sink for report verification. It is similar to SEF and DEF.

The interleave hop-by-hop authentication (IHA)[7] scheme, which is also based on fixed path routing, suffers similar limitations as in CCEF. It needs interleaved upper and lower associations between the sensor nodes to share sensor secrets. Due to the constantly changing and unreliable nature of WSNs, it is implausible to have determined routes. Moreover, these associations demand global knowledge which is complex and costly task for energy-scarce WSNs.

Each node in the bandwidth-efficient cooperative authentication (BECAN) [8] scheme requires a fixed number of neighbors for authentication. It disseminate the authentication keys of en-routing to all sensor nodes in the path. Bit compression is used to save bandwidth. BECAN cannot counter false routing information and selective dropping attacks. Moreover, this novel scheme cannot counter selective dropping and false routing information along with some limitations exhibited by the above schemes.

Greedy perimeter-based stateless routing (GPSR) [9] was generally used in en-route filtering schemes which relied on its simple and robust approach. However, this routing scheme was designed for ad hoc networks and did not performed well for WSNs. It selected a forwarding node among its candidate nodes which is closest to the BS. In case it could not find a path around the perimeter, a right hand rule is employed. The authors of Reference [43] present multipath routing to mitigate improvements in network security including eavesdropping or modification attacks. This work proposed that multipath routing is effective against this type of attack. Furthermore, loading balancing work has been proposed using opportunistic routing [44].

In order to measure energy consumption, the first-order radio model was used [11]. Moreover, the parameters used in the performance measurement of the first-order radio model are adapted from it. An acceptable energy-to-noise ratio is presented in Reference [12]. A lot of works, for instance, Reference [13], has been done to increase network lifetime by improving underlying routing schemes. There is a need to improve network lifetime in security schemes which often ignore routing to prolong network lifetime.

The authors of References [14,15] illustrated the current state of research review on WSNs in general. In a survey [16] on detection and countermeasures against Distributed Denial of Service (DDoS). attacks in WSNs was presented in detail. These attacks waste scarce energy but also cause packets to drop within the network. This proposed scheme can detect fabricated messages and defend against these attacks.

Attacks and countermeasures in sensor networks are discussed in detail in Reference [17]. Reference [18] described recent en-route filtering techniques in WSNs. In the past, it had been observed that unbalanced communication loads in static sink-based networks resulted in the energy-hole problem [21]. This issue exhibited degrading effects on network lifetime.

Simulation experiments are supposed to have Mica2 [22] as experimental platforms in this work. Sensor clocks are synchronised using the synchronization component presented in the energy-efficient time synchronization protocol (ETSP) [23]. Sensor nodes could determine their location using some localization component. For example, in Reference [45], the authors presented a cooperation-based network localization approach.

In References [10,20,26,27,28,29,30,31], the authors proposed their scheme to enhance network lifetime in WSNs. Reference [24] presents a countermeasure for false data filtering in WSNs. A key-indexed-based routing method [25] for filtering false event reports in WSNs is proposed. Several works such as References [10,28,29,30,31] used various methods to extend network lifetime. References [32,33,34] proposed to improve network lifetime and energy efficiency. The authors of References [35,36,38] focused on energy efficiency and that of References [40,41,42] focused on privacy using various techniques.

## 3. Proposed Work

In this section, we present the scheme motivation, problem statement, and implementation details of the proposed AEF scheme.

### 3.1. Motivation

En-route filtering schemes such as DEF, SEF, CCEF, STEF, IHA, and BECAN save energy by countering false report attacks. These schemes often ignore underlying routing, which is based on shortest path routing, resulting in fixed paths. Energy-aware dynamic routing will help extending network lifetimes by distributing communication loads to larger groups of sensor nodes while mitigating the energy-hole problem.

These schemes often exhibit fixed detection capacity, which does not correspond to variations in attacks. By selecting verification nodes (i.e., nodes selected to report validation and to contain a different number of keys) corresponding to current attacks or the false traffic ratio (FTR), our scheme can offer matching dynamic attack responses.

### 3.2. Problem Statement

Network lifetime, security, and energy efficiency are the top three research topics in WSNs. However, WSN schemes often deal with one of these aspects while ignoring the others. Therefore, improving network lifetime while maintaining detection capacity and energy efficiency is an interesting problem. Moreover, a lot of work exists, which rely on routing protocols to extend network lifetime. In en-route filtering schemes, underlying routing is ignored while focusing on the security paradigm. There is a greater need to extend network lifetime in en-route filtering schemes by improving routing and by responding to attacks according to attack intensities with constraints of security and energy.

### 3.3. Network Initialization

At the network setup phase, each node is assigned a unique ID and node key. Sensor nodes are initialized with a fixed amount of energy and sensor range, with some perturbation to represent physical characteristics.

### 3.4. Key Distribution

In the proposed method, the BS can send a query message to an area of interest (i.e., a cluster where we need to enquire an event). This message contains a cluster head (CH) ID, representing that area of interest where a user wants to enquire about an event such as enemy tank movement. To establish communication between the CH and the BS, a key pre-deterministically disseminates en-route nodes on a path established by underlying routing. Since a different number of keys is pre-deterministically distributed on sensor nodes, multiple paths with a different number of keys are possible. Therefore, based on current FTR, we can use paths corresponding to current attacks. For more attacks, a node with more keys can be selected. This capability grants our proposed method the ability to dynamically select paths based on current FTR.

### 3.5. Verification Node Selection

The fitness evaluation function $V_{n}$ for verification node selection is heuristically calculated based on the simple sum of number of node MACs, residual energy, and current false traffic ratio (FTR). The nodes with the highest fitness values among candidate nodes (i.e., nodes on a path) can participate in false report detection using Equation (1).

(1) $$V_{n}=MAC_{n}\times \alpha +Energy_{r}\times \alpha +FTR\times \left(\left(1-\beta \right)/2\right)$$

where α and β are system design parameters. $MAC_{n}$ is the node MAC, and $Energy_{r}$ is the residual energy.

### 3.6. Route Setup

**Figure 2:**
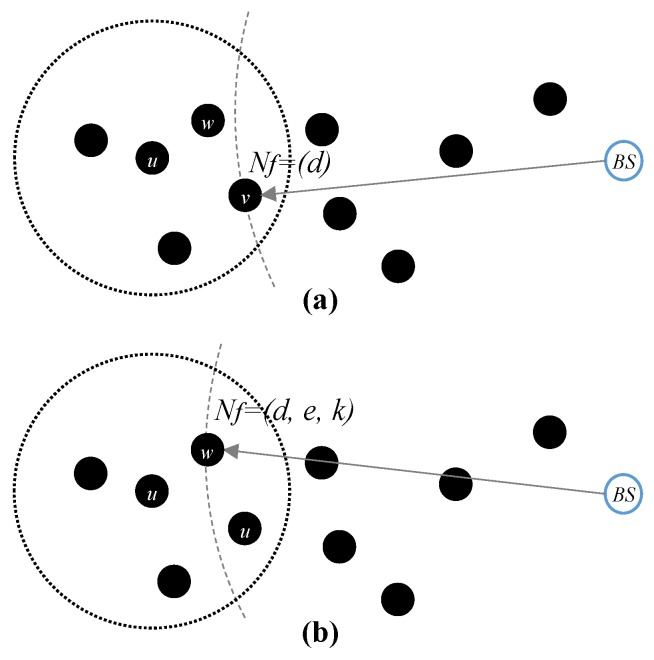
Route setup: (**a**) a conventional shortest path routing and (**b**) energy-aware routing.

Fixed path and energy-aware and dynamic routing in the proposed scheme are shown in Figure 2. The fixed path greedy routing considers only distance, while the proposed routing also caters for residual energy levels and the number of keys on candidate nodes. Following, the fitness evaluation function $N_{f}$ is used to select the next forwarding node (i.e., next mode in the path) with the highest fitness value given in Equation (2).

(2) $$N_{f}=Energy_{r}\times \alpha +Dist_{s}\times \alpha +Keys_{n}\times \left(\left(1-\beta \right)/2\right)$$

where $Dist_{s}$ is the distance of a node from the BS and $Keys_{n}$ is the node key. The path creation is initiated at the BS when it needs to determine an event in an area of interest. This process is repeated to create a path from the BS to the source CH.

#### Example of Energy-Aware Routing

A shortest path or greedy routing used in GPSR [9] is shown in Figure 2a. A forwarding node $N_{f}=v$ is selected among the neighbors of *u* which is the closest to the BS. In the proposed energy-aware dynamic en-route selection routing shown in Figure 2b, we use the fitness function presented in Equation (2). It considers residual energy, closest distance neighbor from the sink, and the number of keys on candidates nodes. A node with the leading value among candidate nodes is selected as the forwarding node, e.g., $N_{f}=w$. The selection of node *w* in energy-aware routing is better than *v* in shortest path routing since the former considers the energy levels of a node and keys.

### 3.7. Energy Consumption Model

**Figure 3:**
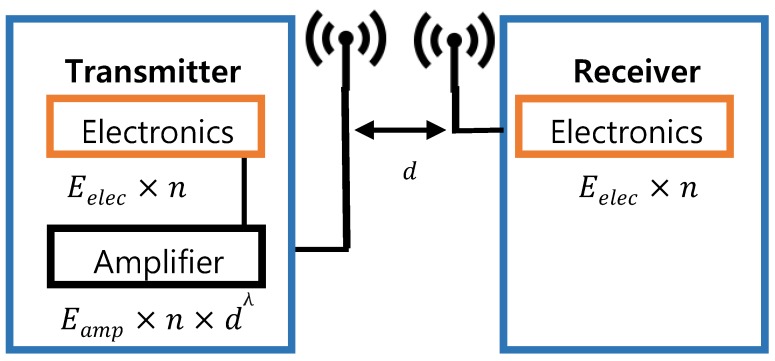
First-order radio model for energy consumption analysis.

For energy consumption analysis, the first-order radio model is used, having a free ($d^{2}\;$power loss) channel model. We only cater for energy dissipation related to radio components and circuits of sensor motes as illustrated in Figure 3. For the transmission of a kbits message carried to a *d* distance between the transmitter and receiver radios, the transmission energy necessary $E_{T_{x}}\left(k,\;d\right),$ is given in Equation (3).

(3) $$E_{T_{x}}\left(k,\;d\right)=E_{elec}\times k+E_{amp}\times k\times d^{\lambda }$$

In Equation (4), $E_{elec}$ is the energy consumed by the circuit electronics and $E_{elec}\times n$ is the energy required by the transmitter electronics to transfer *k* bits. Additionally, the energy required by the amplifier is $E_{amp}$ and the path loss constant is represented by λ. Similarly, $E_{R_{x}}\left(n\right)$ indicates the required energy in *k* bits as follows:

(4) $$E_{R_{x}}\left(k\right)=E_{elec}\times k$$

$\;E_{amp}=100pJ/bit/n^{2}$ is the transmitter amplifier energy for transmission. Moreover, 50nJ/bit is the energy consumption by transmitter and receiver circuits. Assumptions related to radios such as energy of transmission and receptions for single bit will change the advantages of different protocols. Therefore, the above-selected acceptable energy-to-noise ratio could avoid otherwise biased values. The optimal values of $E_{elec}$ and $E_{amp}\;$ are chosen from Reference [12] to attain an acceptable $E_{b}/N_{0}$. There is no hardware changes required to deploy this model on sensor nodes. An energy and network lifetime analysis of the first-order radio model is presented [11].

### 3.8. Energy-Hole Model and Discussions

In static sink-based WSNs, events or messages are replayed to the BS using a multihop many-to-one communication model. This results in uneven energy consumption loads. The energy-hole problem mathematical description is discussed in this section. Based on the energy-hole problem presented in Reference [46], we present a modified mathematical model for semi-rings where the BS is located at the middle bottom of the sensor field.

**Figure 4:**
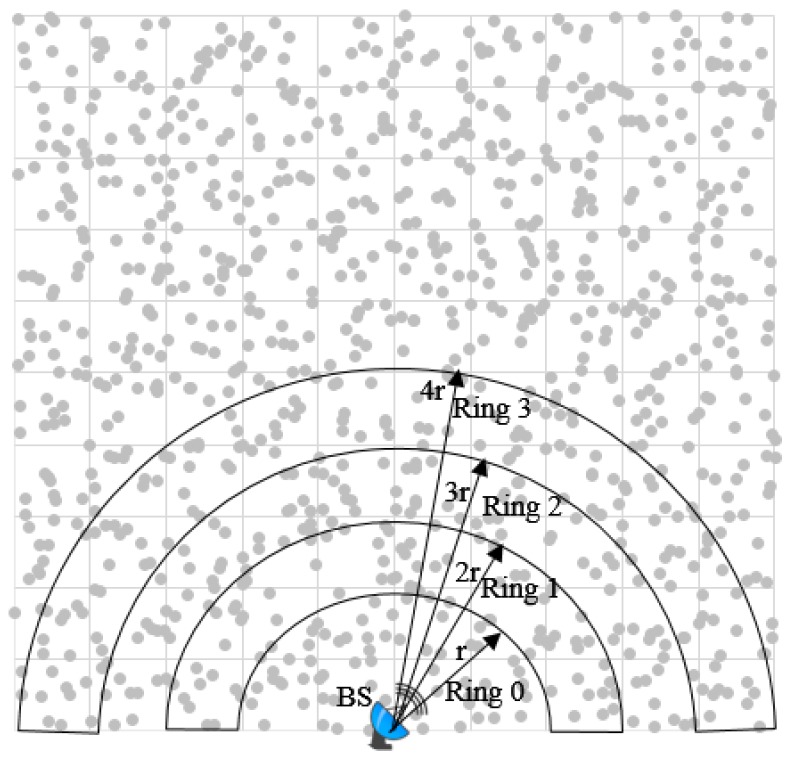
Energy-hole problem due to a faster rate of energy consumption around the base station (BS) and critical paths.

A square sensor field of uniformly and randomly deployed sensors in an area of $F_{w}\times F_{h}\;=\left(F\times F\right)\;m^{2}$ is shown in Figure 4, where F=M×r meters. In a uniformly and randomly deployed sensor network, the node density is uniform in the entire network:

(5) $$p=\frac{N}{A_{sn}}$$

where *N* is the total number of sensor nodes and $A_{sn}$ is the coverage area of the sensor network. BS is located at the bottom center of the sensor field; the whole area can be divided into $\frac{M}{2}$ bands with step sizes of *r* meters. The ring 0 is a small semi-ring of radius *r* meters, and ring 1 has a radius of 2*r* meters and so on. We assume that messages can move from one ring to the next using one hop for simplicity, although in practice, in order to cross a single ring, multiple hops may be traversed. It can be observed that most communication load frequencies are on nodes around the BS and on critical paths as compared to nodes farther from the BS. Inner rings (in particular, ring 0, as it needs to relay all outer rings data to the BS in addition to replying ring data) also need to transmit their own messages. Therefore, intuitively, the rate of energy consumption load on rings closer to the BS is faster as compared to outer rings. Thus, as the ring 0 node’s energy level reaches zero, the BS cuts off from the entire network, known as the energy-hole or hot-spot problem. Thus per node communication load (ł) in semi-ring zero ($s_{ring0}$) that is half of the load in full ring is illustrated by Equation (6).

(6) $${ł}_{s_{ring}0}=\frac{1}{2}\left(\frac{total\;network\;traffic}{number\;of\;nodes\;in\;s_{ring0}}\right)=\frac{\rho {\left(Mr\right)}^{2}b}{2\rho \pi r^{2}}=\frac{M^{2}}{2\pi }b$$

Similarly, for $s_{ring1}$, per node communication load is given by Equation (7)

(7) $${ł}_{s_{ring1}}=\frac{1}{2}\left(\frac{total\;load\;from\;outside\;s_{ring}0}{number\;of\;nodes\;in\;s_{ring0}1}\right)=\frac{\left(\rho {\left(Mr\right)}^{2}-\pi r^{2}\right)b}{2\rho \left(\pi r^{2}-\pi r^{2}\right)}=\frac{\left(\frac{M^{2}}{\pi }-1\right)}{6}b$$

and more generally, the $i_{th}$ semi-ring load can be represented by Equation (8)

(8) $${ł}_{s_{ring\;i_{th}}}=\frac{\left(\rho {\left(Mr\right)}^{2}-\pi \left(ir^{2}\right)\right)b}{2\rho \left({\pi \left(i+1\right)r)}^{2}-\pi {\left(ir\right)}^{2}\right)}=\frac{\left(\frac{M^{2}}{\pi }-i^{2}\right)}{2(2i+1)}b$$

where $i=0,1,\ldots ,(\frac{M}{2}-1)$.

There is considerable difference in energy consumption in different semi-rings as can be observed from the above mathematical model. With proceeding inner rings, the rate of energy consumption increases around the BS.

### 3.9. False Report Attacks Information

In this section, we explain our method that can calculate the current attacks ratio or false traffic ratio (FTR) without causing extra messages or energy costs at the sensor nodes.

The communication is based on a query-driven model. The BS can send a query to determine an event in a a target area. The corresponding CH in the area along with event sensing nodes collaborate to respond. For one such communication, the BS has information for the number reports from CH in the area of interest.

A validated event report arriving at the BS will cause increments in the legitimate report counter by one. Here, additional messages or energy consumption is not needed. A false report can be dropped either at the BS or en-route. When a false report is dropped at the BS, the false report counter is incremented by one. In case it is dropped at one of the nodes on the path, the BS will know it, as it will not be received within the time window. Therefore, using these two counter information available on the BS, FTR for *m* number of events in the WSN can thus be calculated as follows:

(9) $$FTR=\frac{\sum_{i=1}^{m}\left(1\times F_{i}\right)}{\sum_{i=1}^{m}\left\{1\times F_{i}+1\times L_{i}\right\}}$$

where $F_{i}\in \left[0,1\right]$ and $L_{i}=F_{i}^{\prime }$ indicate the fabricated and legitimate reports, respectively, defined as follows:

(10) $$F_{i}=\left\{{}_{0\;\mathrm{if}\;\mathrm{the}\;i_{th}\mathrm{report}\;\mathrm{is}\;\mathrm{valid}.\;}^{1\;\mathrm{if}\;\mathrm{the}\;i_{th}\mathrm{report}\;\mathrm{is}\;\mathrm{false}.\;}\right.$$

In Equation (9), *i* represents the current number of events from 1 to *m* (i.e., total number of events in WSN). Fabricated reports are false reports injected from a compromised node in control of an adversary. Legitimate reports are valid reports generated by non-compromised nodes in WSN. The counters are at the BS, which has sufficient power. Therefore, we can model current attack information without costing extra messages or energy.

## 4. Experimental Results

### 4.1. Experimental Setup

#### 4.1.1. Assumptions

The BS cannot be compromised while sensor nodes are considered secure in the network initialization phase. The sensor field, sensor nodes, and the BS are assumed to be stationary. The BS knows the IDs and node keys of all the sensors. In the first-order radio model, we only considered the energy emancipation related to radios. We also assumed that the underlying sensor platform for our simulation experiments is a MICA2 sensor mote [22]. Each sensor node’s system clocks are synchronized using a time synchronization mechanism [23]. Sensor nodes can determine their location using some localization components [45].

#### 4.1.2. Simulation Environment

Simulation experiments were performed in a custom built C++ simulator.

Each node is assigned a unique ID and node key before random deployment.

**Figure 5:**
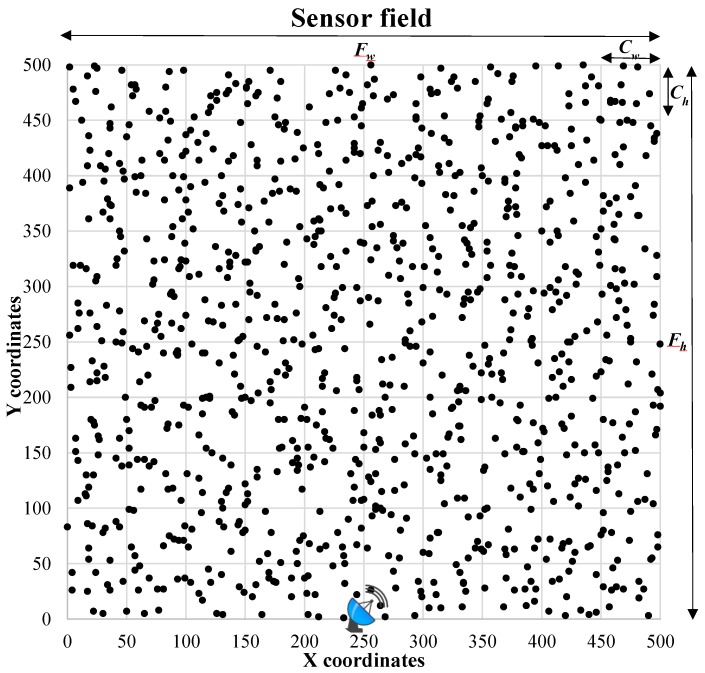
Square sensor field with the BS at the bottom center.

Simulation parameters are shown in Table 1. In order to represent variation in the range of sensors in a physical environment, a ±10% perturbation is introduced. Moreover, energy in batteries is not fixed; therefore, ±5% perturbation is randomly exhibited here as well. The sensor field setup is illustrated in Figure 5. The location of the sensor nodes is represented by *x* and *y* coordinates. The art of the square field is $\left(F_{w}\times F_{h}\right)\;m^{2}$ consisting of square clusters with area $\left(C_{w}\times C_{h}\right)\;m^{2}$ each. The BS is located at the lower middle of the sensor field. The sensor field is composed of randomly deployed *n* number of sensors, where *n* = 400, 700, and 1000. Each cluster consists of the same number of sensor nodes.

### 4.2. Performance Evaluation

#### 4.2.1. Performance Measurement

A square sensor field of $\left(500\times 500\right)\;m^{2}$ consists of three scenario with 400, 700, and 1000 nodes. Furthermore, with regard to false report attacks, two cases of FTRs of 50%, 70%, and 90% are considered for each of the above scenarios. The BS is located at bottom center of the sensor field. The sensor range, cluster height, and width are 50 m. Initial node energy of each sensor node is 1 joule with 5% perturbation and 10% fluctuation in the sensor range to reflect physical conditions. One round of communication consists of 128 bytes of data received at the BS. For each simulation experiment, 100 iterations have been carried out.

Network lifetime is measured using first cut-off (FCO), which is the number of rounds required to consume all energy of a sensor node, i.e., first sensor node is depleted. Moreover, 10 percent of nodes depleted (10PCO) and last cut-off (LCO) are also used to measure the network lifetime. 10PCO is 10% of the total number of nodes depleted. The LCO performance metric is defined as the total number of nodes depleted when there is no more communication possible between the BS and sensor nodes. Energy efficiency is measured for the average energy consumed per round. Detection capacity is defined as the number of attacks actually detected (AD) divided by total attacks (TA), as shown in Equation (5).

(11) $$DP=\frac{AD}{TA}$$

In the proposed method, the forwarding node from candidates nodes is selected based on the number of keys corresponding to the current FTR. A total of nine cases are considered for network lifetime using three FTR and over three performance metrics. Three cases for each energy-efficiency and detection capacity are illustrated.

#### 4.2.2. Network Lifetime Analysis

In dynamic path routing, as against fixed path routing, multiple paths are created. In fixed path routing, a communication is carried out through a single path as long as that path exists. That path is as good as the first node on that path is depleted due to the energy level reaching zero a after certain number of communications through that path, let us say *n*. Since, in dynamic path routing, *k* number of paths can be chosen in the case of dynamic path routing with kn networks lifetime, this means *k* times network lifetime can be extended. For example, if each have 1 joule of energy and one communication costs 0.1 joules of energy consumption on each node, then network lifetime of that path is *n* = 10 events. In case there are *k* = 3 paths available, which can be chosen alternatively, then the upper limit of network lifetime extension is 3 times as compared to fixed path routing, which is significant.

#### 4.2.3. Cost Analysis

The main contributing factors towards total cost of proposed scheme include (1) pre-deterministic key distribution, (2) dynamic path routing, and (3) current attack information methods. As illustrated in Section 3.8, item (3) does not need any extra messages on the sensor nodes, so it does not contribute towards the cost of solution. Key distribution is performed at the initialization phase and does not incur cost towards performance evaluation. Dynamic routing method (2) is different from the fixed path routing as the former considers multiple parameters to select the next node as explained in route setup in Section 3.6. The relevant cost of these methods is trivial and have been included in performance measurements.

### 4.3. Performance Results

In this section, details of the performance measurement metrics and simulation experiment results are illustrated.

#### 4.3.1. Network Lifetime

**Figure 6:**
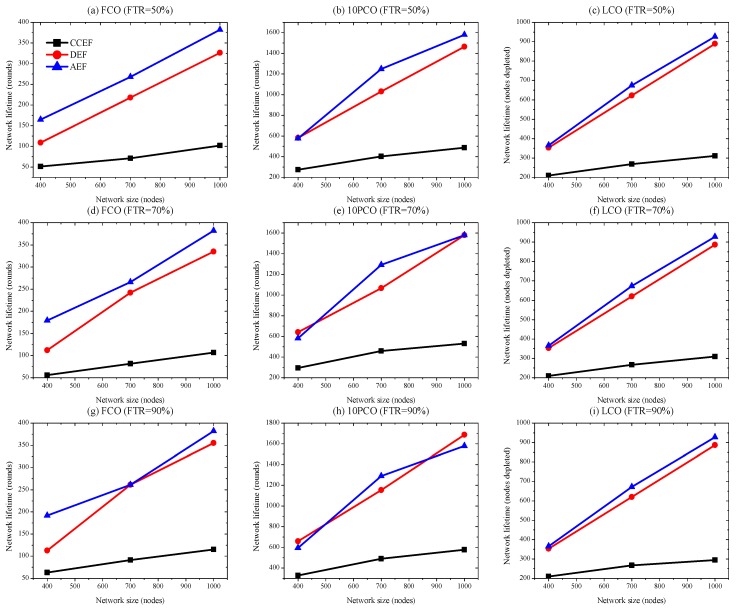
Network lifetime: six simulation experimental setups using FCO, 10PCO, and LCO for 50% and 70% false traffic ratios (FTRs) for network sizes of 400, 700, and 1000 nodes.

The network lifetime performance of AEF is shown in Figure 6. The *x*-axis represents the network size in terms of the number of sensor nodes and the *y*-axis illustrates the number of rounds. The summary of network lifetime improvement over CCEF and DEF is illustrated in Table 2. It can be observed that relative improvements decreases as the number of rounds or attacks increases.

- **FCO:** AEF outperforms CCEF by 2.92 times or 292% for the FCO performance parameter at 50% FTR as shown in Figure 6a. For 70% and 90%, FTR network lifetime also have significant improvements of 2.81 folds and 2.71 times, respectively, as illustrated in Figure 6b,c. In comparison with DEF, we observe relatively lower improvements of 1.25, 1.20, and 1.15 times with respect to 50%, 70%, and 90% FTR.
- **10PCO:** AEF shows a gain of 2.64, 2.73, and 2.74 times against CCEF at 50%, 70%, and 90% FTR, respectively. In case of DEF, there is also relativity small marginal improvements of 1.11 and 1.05 times at 50% and 70% FTR, repectively. However, AEF shows little decrease in network lifetime at 90% FTR.
- **LCO:** Performance improvement over the number of rounds required for the last node to be depleted for 50% FTR is 2.36, for 70% FTR is 2.37, and for 90 % is 2.41 folds in comparison with CCEF. In the case of DEF, marginally less improvements of 1.05 (50% FTR), 1.06 (70% FTR), and 1.06 (90% FTR) times are observed.

It can be observed that, with an increase in the number of the FTRs or the rounds, the relative improvement in network lifetime decreases. However, there is no set trend; however, the larger the number of rounds for a small network size, the more the margin of improvement decreases. This trend by part is because of random deployment, different network sizes, and dynamic path selection. It can be observed from Table 2 that there is significant improvement against CCEF. There is also network lifetime gain against DEF; however, the margin of improvement is less. The reason is because DEF considers energy-aware routing.

#### 4.3.2. Energy-Efficiency

**Figure 7:**
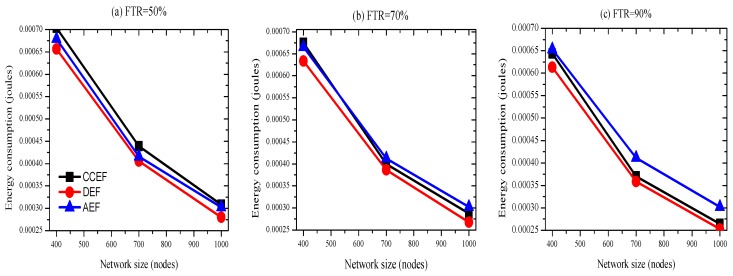
Energy efficiency (FTR 50, 70, and 90% at first cut-off (FCO)).

The *x*-axis in Figure 7 represents the network size in term of the number of nodes, and the *y*-axis shows energy consumption per round in joules. We observe that, for the average energy consumed per round, performance metrics among CCEF, DEF, and AEF show similar energy consumption. The trivial increase in energy cost of the proposed scheme is due to energy-aware routing instead of using greedy or shortest path routing. Our method also considers current attack information to respond accordingly. However, our scheme performs better as compared to CCEF in energy efficiency. At large, energy efficiency demonstrates similar performances. Moreover, the energy consumption per round decreases for all schemes as the network size increases. On average, energy consumptions for CCEF, DEF, and AEF are $4.55^{-4}$, $4.32^{-4}$, and $4.60^{-4}$. A summary of detection capacity comparison is shown in Table 3. Considering negative exponents and significant increases in network lifetime, we can state that the energy efficiencies have similar performances.

#### 4.3.3. Detection Capacity

**Figure 8:**
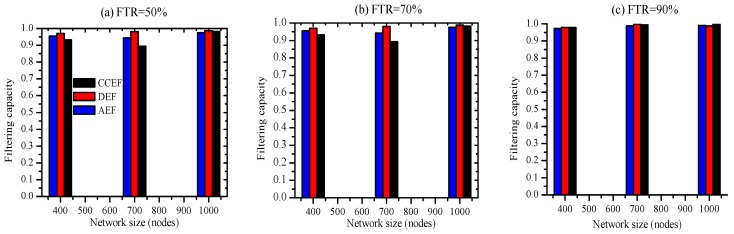
Detection capacity (FTR 50, 70, and 90% at FCO).

In Figure 8, the *y*-axis represents the detection capacity ratio while the *x* axis illustrates the three different network setups of sizes with 400, 700, and 1000 nodes. We can observe that the detection capacity of AEF on average is similar to that CCEF and DEF for 50% FTR. For any FTR, the marginal performance of each scheme exhibits similar trends. The detection capacities for CCEF, DEF, and AEF are 99.00%, 98.47%, and 98.31, respectively. Therefore, with trivial difference, we can claim that detection capacity is maintained. A summary of energy-efficiency is presented in Table 4.

## 5. Conclusions

The proposed AEF scheme demonstrates significant improvements over network lifetimes while maintaining detection capacity and energy efficiency. As false report attacks increase, the relative margin of gain in network lifetime lowers. Moreover, lifetime over a large number of rounds (i.e., FCO, 10PCO, and LCO) decreases the relative margin of improvement. Generally with an increase in FTR, a smaller network size, and an increase in number of rounds, we observe less improvements in network lifetime; however, this is not a fixed trend.

In AEF, we used different methods to increase network lifetime which resulted in even distribution of energy consumption loads over the larger group of participating sensor nodes. It can dynamically give matching responses according to the current attack ratio. We calculated current FTR without causing extra messages on sensor nodes. Further, it can be stated that, by carefully designing en-route filtering schemes, not only effective filtering but also network lifetime gain can be achieved.

## 6. Future Work

In the future, we plan to use fuzzy logic systems for selecting filtering nodes. As static sink-based networks suffer from the energy-hole problem, another approach to counter this problem is mobile sink. It will not only further minimize the energy-hole problem but also improve network lifetime. As the density of sensor nodes decreases in a cluster, it increases the probability of network partitioning. In order to counter this problem, a network density-based re-clustering approach can be instrumental in this regard. 

References

## Figures and Tables

**Table 1:** Simulation parameters.

Parameters	Values
Sensor field size	$\left(500\times 500\right)\;m^{2}$
Cluster size	$\left(50\times 50\right)\;m^{2}$
BS location	(250,0)m
Sensors nodes	400, 700, 1000
$R_{i}$	50 m (±10%)
$E_{elec}$ for Tx and Rx	50 nJ/bit
$E_{amp}$	100 pJ/bit/m${}^{2}$
Node energy	1 Joules (±5%)
MAC verification	20 mJ
Data packet	32 bytes
Round	128 bytes
FTR	50% , 70%, 90%
Path loss constant (λ)	2

**Table 2:** Network lifetime gain of adaptive en-route filtering (AEF; times) over commutative cipher-based en-route filtering (CCEF) and dynamic en-route filtering (DEF).

FTR	FCO	10PCO	LCO	Avg.
CCEF				
50%	2.92	2.64	2.36	2.64
70%	2.81	2.73	2.37	2.63
90%	2.71	2.74	2.41	2.61
DEF				
50%	1.25	1.11	1.05	1.14
70%	1.20	1.05	1.06	1.10
90%	1.15	0.95	1.06	1.05

**Table 3:** Energy efficiency comparison (joules).

FTR	CCEF	DEF	AEF
50%	$4.84^{-4}$	$4.47^{-4}$	$4.66^{-4}$
70%	$4.54^{-4}$	$4.29^{-4}$	$4.59^{-4}$
90%	$4.26^{-4}$	$4.19^{-4}$	$4.55^{-4}$
avg.	$4.55^{-4}$	$4.32^{-4}$	$4.60^{-4}$

**Table 4:** Detection capacity comparison (% age).

FTR	CCEF	DEF	AEF
50%	93.64	97.96	95.80
70%	96.65	97.64	96.75
90%	99.00	98.47	98.31
avg.	99.00	98.47	98.31
